# Phase I clinical study of brentuximab vedotin (SGN-35) involving children with recurrent or refractory CD30-positive Hodgkin’s lymphoma or systemic anaplastic large cell lymphoma: rationale, design and methods of BV-HLALCL study: study protocol

**DOI:** 10.1186/s12885-018-4042-1

**Published:** 2018-02-01

**Authors:** Masahiro Sekimizu, Akihiro Iguchi, Tetsuya Mori, Yuhki Koga, Akiko Kada, Akiko M. Saito, Keizo Horibe

**Affiliations:** 1 Department of Pediatrics, National Hospital Organization Nagoya Medical Center, Nagoya, Japan; 2 Department of Pediatrics, Hokkaido University Hospital, Sapporo, Japan; 3 Department of Pediatrics, St. Marianna University School of Medicine Hospital, Kawasaki, Japan; 4 Department of Pediatrics, Kyushu University Hospital, Fukuoka, Japan; 5 Clinical Research Center, National Hospital Organization Nagoya Medical Center, Nagoya, Japan

**Keywords:** Brentuximab vedotin, SGN-35, Children, Hodgkin’s lymphoma, Anaplastic large cell lymphoma

## Abstract

**Background:**

Hodgkin’s lymphoma (HL) and anaplastic large-cell lymphoma (ALCL) are the two most common tumors expressing CD30. Internationally, a clinical study that is being conducted involving adults with recurrent or refractory HL or ALCL suggests efficacy of brentuximab vedotin (SGN-35). Pediatric patients should be given medicines that have been appropriately evaluated for their use. In the past, however, new approved drugs have been used for pediatric patients without the confirmation of safety and efficacy in pediatric patients. Therefore, it is important to examine the safety and efficacy of SGN-35 in Japanese children.

**Methods:**

Phase I clinical study of SGN-35 involving children with recurrent or refractory CD30-positive Hodgkin’s lymphoma or systemic anaplastic large cell lymphoma (BV-HLALCL study) is being conducted for pediatric patients in order to evaluate the safety, feasibility and preliminary clinical effectiveness of brentuximab vedotin. SGN-35 is intravenously administered on Day 1 of each cycle (21 days/cycle). The dose of SGN-35 is calculated based on the body weight at the baseline. The primary endpoint is dose limiting toxicity and incidence of adverse events. The secondary endpoints are pharmacokinetics, response rate, complete remission rate, response duration, progression-free survival and event-free survival. The reduction rate of tumor will be calculated according to revised response criteria for malignant lymphoma for measurable tumor. Six pediatric patients will be enrolled in this study.

**Discussion:**

This study aims to expand indication of SGN-35 in Japan by assessing its safety and efficacy in pediatric patients.

**Trial registration:**

JMACCT ID: JMA-IIA00229. Registered on 17 Nov 2015.

## Background

Hodgkin’s lymphoma (HL) and anaplastic large-cell lymphoma (ALCL) are the two most common tumors expressing CD30. The treatment of HL and ALCL has largely relied on cytotoxic chemotherapy.

Basic treatment for childhood HL consists of chemotherapy and low-dose involved field radiotherapy (LD-IFRT). Chemotherapy alone or a combination of chemotherapy and LD-IFRT is selected in accordance with individual children. Furthermore, the intensity of initial chemotherapy is determined based on early treatment responsiveness in order to avoid unnecessary additional chemotherapy or radiotherapy. Chemotherapeutic regimens and treatment schedules differ among clinical studies. In Japan, treatment has not been standardized, and is selected based on the results of international clinical studies in accordance with individual patients. In a representative clinical study regarding childhood HL, the GPOH-HD-2002 study, chemotherapy with vincristine, etoposide, prednisolone, doxorubicin, cyclophosphamide, and procarbazine and LD-IFRT for some patients improved the 5-year event-free survival rate to 89%, and the 5-year survival rate to 97% [1]. In Japan, this treatment is selected for many patients. Treatment for patients with treatment resistance/relapse, accounting for approximately 10% of those with childhood HL, has not been standardized. Patients with local relapse after initial treatment for a low-risk group may be saved by chemotherapy and LD-IFRT, but the exacerbation-free survival rate ranges from 30 to 65% in other patients with treatment resistance/relapse even when hematopoietic cell transplantation is performed [2, 3].

As standard treatment for childhood ALCL, ALCL99, of which the efficacy and safety were confirmed in an international cooperative clinical study involving Europe and Japan, is selected. It refers to combination chemotherapy with dexamethasone, cyclophosphamide, high-dose methotrexate, ifosfamide, etoposide, cytarabine, and doxorubicin. In 352 patients enrolled in the study, the 2-year event-free survival rate was 74.1%, and the 2-year survival rate was 92.5% [4]. There were no marked differences in the results among countries participating in the clinical study. Although the results of initial treatment for childhood ALCL are favorable, it is necessary to arrange treatment for a high-risk group (proportion: approximately 20%) and patients with relapse (proportion: approximately 30%). Retrospective studies suggest the efficacy of allogeneic hematopoietic stem cell transplantation for treatment-resisting patients with progression early after the start of initial treatment and those in whom relapse is frequently detected despite their responses to chemotherapy [5, 6]. Furthermore, another study suggests the efficacy of monotherapy with vinblastine for patients with relapse [7]. However, an optimal treatment period has not been established, and long-term treatment is conducted in many cases.

Although the results of initial treatment for childhood HL and ALCL are favorable, it is necessary to arrange treatment for patients with relapse or refractory. Targeted lymphoma therapy, using an anti-CD30 antibody, provides an innovative treatment modality for specific lymphomas, particularly HL and ALCL.

Brentuximab vedotin (SGN-35) is a new antibody-drug conjugate (ADC) that binds to a cell surface marker, CD30, manufactured by Seattle Genetics, Inc. (SG, Inc.). CD30 is a type 1 membrane-penetrating protein, belonging to the tumor necrosis factor receptor super family. It appears on the Reed-Sternberg cells of HL patients and T cells of those with ALCL or other T-cell-mediated lymphoproliferative diseases.

SGN-35 consists of 3 components: (i) anti-CD30 monoclonal antibody (cAC10), (ii) a potent microtubule inhibitor, monomethylauristatin E (MMAE), and (iii) a linker decomposed by protease. A covalent bond between cAC10 and MMAE is mediated by this linker. The biological activity of SGN-35 appears through the following steps: initially, when SGN-35 binds to CD30 on the tumor cell surface, it is transported to lysosome through intracellular uptake as ADC-CD30 complex. Subsequently, MMAE is released in the intracellular area through protein-decomposing reactions. MMAE binds to tubulin, destroying an intracellular microtubular network and inducing the arrest of the cell cycle. As a result, apoptosis of CD30-expressing tumor cells occurs.

Internationally, a phase I study of SGN-35 (SG035–0001 study) involving patients with recurrent or refractory CD30-positive hematopoietic organ tumors was conducted by SG, Inc. from November 2006 [8]. Thereafter, a phase II study (SG035–0003 study) involving patients with recurrent or refractory CD30-positive HL after autologous hematopoietic stem cell transplantation was conducted from February 2009 [9], and another phase II study involving patients with recurrent or refractory CD30-positive systemic ALCL (sALCL) (excluding those with primary dermal ALCL localized in the skin)(SG035–0004 study) was performed from June 2009 [10]. SGN-35 was approved as ADCETRIS (proprietary name) to be indicated for HL and sALCL patients in August 2011 in the United States and in October 2012 in European Union.

In Japan, a phase I/II study (TB-BC010088 study) involving patients with recurrent or refractory CD30-positive HL or sALCL was conducted by Takeda Bio Development Center Limited from October 2011 [11]. Based on the results of the TB-BC010088, SG035–0003, and SG035–0004 studies as the main studies, a new drug application of SGN-35 was submitted by Takeda Pharmaceutical Company Limited. SGN-35 (proprietary name: ADCETRIS) was approved for patients with recurrent or refractory CD30-positive HL or ALCL. The administration method/dosage is as follows: for adults, brentuximab vedotin (gene recombinant) at 1.8 mg/kg (body weight) should be intravenously infused every 3 weeks. If necessary, the dose should be decreased in accordance with the patient’s condition.

Clinical studies involving adults with recurrent or refractory HL or ALCL in Japan and other countries demonstrated the efficacy and safety of SGN-35. Internationally, a clinical study that is being conducted involving adults with recurrent or refractory HL or ALCL suggests its efficacy. Pediatric patients should be given medicines that have been appropriately evaluated for their use. In the past, however, new approved drugs have been used for pediatric patients without the confirmation of safety and efficacy in pediatric patients. Therefore, it is significant to examine the safety and efficacy of SGN-35 in Japanese children.

In TB-BC010088 study involving Japanese adults [11], dose escalation was started from 1.2 mg/kg. In this dose level, no dose-limiting toxicity (DLT) was observed. In C25002 study involving non-Japanese children [12], dose escalation was started from 1.4 mg/kg. In this dose level also, no DLT was observed. From these results, we omitted dose escalation started from lower than 1.8 mg/m2. The maximum tolerance dose (MTD) of this drug was clarified as 1.8 mg/kg with good efficacy in SG035–0001 clinical study involving adult patients [8]. Therefore, we omitted consideration for more than 1.8 mg/kg dose. From the above, we adopted the design considering only a single dose of 1.8 mg/kg in this study.

## Methods/design

### Protocol digest of the study

#### Objectives

Primary objective is to examine the safety and tolerance of SGN-35 in children with recurrent or refractory CD30-positive HL or sALCL. Accessory objective is to investigate the pharmacokinetics and efficacy of SGN-35 in children with recurrent or refractory CD30-positive HL or sALCL.

#### Study setting and protocol review

This is a single-arm, open-label, multicenter phase I study involving four institutions: Hokkaido University Hospital, St. Marianna University School of Medicine Hospital, National Hospital Organization Nagoya Medical Center, and Kyushu University Hospital. The protocol has been reviewed and approved by institutional review boards of each institutions.

#### End points

<Primary endpoints>

(i) DLT
(ii) Adverse events

<Secondary endpoints>

(i) Pharmacokinetics (serum concentration of SGN-35, plasma concentration of MMAE, and serum concentrations of all antibodies)
(ii) Overall response rate (ORR)

ORR refers to the proportion of subjects with complete remission (CR) or partial remission (PR) as the best comprehensive response in analysis set.

(iii) Complete remission rate

CR rate refers to the proportion of subjects with CR as the best comprehensive response in analysis set.

(iv) Response duration

The response duration refers to a duration from the first day of CR or PR evaluation until the first day of progressive disease (PD) evaluation or the day of death related to some factor (earlier). With respect to data on the response duration in subjects continuing to participate in this study until analysis without PD, those receiving anti-tumor treatment other than the test regimen and stem cell transplantation, and those excluded from this study before CR or PR achievement, the final day of image assessment on which disease progression is ruled out in lesions to be measured is regarded as the day of censoring.

(v) Progression-free survival (PFS)

PFS refers to a period from the first day of administration until the first day of PD evaluation or the day of death related to some factor (earlier). With respect to data on PFS in subjects who continued study participation until analysis without showing PD, those who received anti-tumor treatment other than test treatment and stem cell transplantation, and those excluded from this study before CR or PR evaluation, the last day of image assessment on which disease progression was ruled out in lesions to be measured is regarded as the day of censoring. Subjects in whom image assessment after initial administration was not conducted, is censored at day 1.

(vi) Event-free survival (EFS)

Events include death, progression of disease, secondary cancer, and toxicity-related discontinuation. Toxicity-related discontinuation refers to the discontinuation of this clinical study related to adverse events of which the relationship with this drug cannot be ruled out. EFS refers to the interval from the first day of administration until the earliest day of event appearance. If there are no events, the subject is censored at the final day of observation. If it is discontinued due to transplantation or withdrawal in the absence of events, they are censored at the day of study discontinuation. If subjects receive new anti-tumor treatment other than stem cell transplantation, they are censored at the start of the treatment.

The anti-tumor effects of SGN-35 are evaluated only in patients with measurable lesions according to the Revised Response Criteria for Malignant Lymphoma [13].

#### Eligibility criteria

1. Asian patients aged 2 to 17 years on obtaining informed consent.
2. Those definitively diagnosed with CD30-positive HL or sALCL based on histological findings. A report or its copy describing that a specimen collected at the time of initial diagnosis or relapse was evaluated as positive for CD30 using an immunohistochemical procedure or flow cytometry is stored in the hospital.
3. Those with PD during standard chemotherapy or without CR/partial remission (PR) after treatment, or those with relapse or additional exacerbation after standard chemotherapy.
4. Those with an Eastern Cooperative Oncology Group performance status (PS) of 0 to 2.
5. Those whose laboratory data on screening meet the following criteria. The administration of a gene recombinant human granulocyte-colony stimulating factor (G-CSF) preparation or blood transfusion is not performed within1 week before neutrophil and platelet count tests:

- Neutrophil count: ≥1500 × 10^6^/L
- Platelet count: ≥75,000 × 10^6^/L
- Hemoglobin level: ≥8 g/dL
- Serum bilirubin level: ≤1.5-fold the upper limit of normal (ULN) in the facility
- Serum creatinine level: ≤1.5-fold the ULN
- Alanine aminotransferase (ALT) and aspartate aminotransferase (AST): ≤2.5-fold the ULN

6) Those who are expected to survive for ≥3 months on obtaining informed consent.
7) Written informed consent regarding participation in this clinical study could be obtained from subjects and/or representatives

#### Exclusion criteria

1. Patients diagnosed with primary ALCL of the skin as the latest diagnosis (those with infiltration in other organs and a sALCL-like condition are regarded as eligible).
2. Those after the resection of all lesions.
3. Those with active viral, bacterial, or fungal infection within 2 weeks before the initial administration of SGN-35.
4. Those with ≥grade III heart failure (New York Heart Association (NYHA) severity classification), refractory coronary disease, arrhythmia, a left ventricular ejection fraction of < 50%, angina pectoris, or acute ischemia or active conduction disorder on electrocardiography, or those with a history of myocardial infarction within 6 months before the initial administration of SGN-35.
5. Those with refractory diabetes.
6. Those with a history of other malignant tumors persisting for ≥3 years and complications. However, the following cancers are excluded:

- Completely resected non-melanoma skin cancer
- Completely resected intraepithelial carcinoma

7) Those with intra-cerebral or meningeal infiltration.
8) Those with signs or symptoms suggesting progressive multifocal leukoencephalopathy.
9) Those with a history of severe hypersensitivity or allergy.
10) Human immunodeficiency virus antibody-, hepatitis B virus surface antigen (HBs) -, hepatitis B virus surface antigen antibody-, hepatitis B virus core antigen antibody-, or hepatitis C virus antibody-positive patients on a screening test. However, patients with a history of hepatitis B vaccination who are positive for HBs antibody alone will not be excluded.
11) Patients with liver cirrhosis.
12) Those in whom autologous stem cell transplantation was performed within 12 weeks before the initial administration of SGN-35.
13) Those in whom allogeneic stem cell transplantation was performed.
14) Those who received treatment for malignant tumors (radiotherapy, chemotherapy, and hormonal therapy) within 2 weeks before the initial administration of SGN-35. However, those who received biological preparations in a longer period: either 6 weeks before the initial administration of this drug or a period corresponding to 5-fold the half-life, will be excluded.
15) Those who received the systemic administration of adrenocorticohormones at a non-steady dose within 1 week before the initial administration of SGN-35.
16) Those who took drugs that inhibit CYP3A4 (clarithromycin, itraconazole, verapamil, and diltiazem) or ingested foods/supplements (such as grapefruit) within 1 week before the initial administration of SGN-35.
17) Those who took drugs that induce CYP3A4 (phenytoin, phenobarbital, rifampicin, carbamazepine) or ingested foods/supplements (such as St. John’s wort) within 2 weeks before the initial administration of SGN-35.
18) Those to whom other investigational drugs were administered within 4 weeks before the initial administration of SGN-35.
19) Those in whom medical instruments under a clinical study were used within 4 weeks before the initial administration of SGN-35.
20) Those with hypersensitivity to additives contained in the composition of SGN-35.
21) Pregnant (human chorionic gonadotropin-positive) or lactating patients.
22) Those who are not willing to conduct appropriate contraception from informed consent acquisition until 6 months after the final administration of the investigational drug.
23) Those with positive reactions on a pregnancy test at the time of screening.
24) Those in whom the chief investigator/investigators considered it difficult to perform this clinical study.

### Treatment methods

SGN-35 is intravenously administered on Day 1 of each cycle (21 days/cycle). After Cycle 2, SGN-35 should be administered − 1 to + 3 days from the established day of administration, excluding cases in which a specific duration is required to achieve recovery from toxicity, appearing from the preceding cycle of SGN-35 therapy, of which the association with the investigational drug cannot be ruled out.

The drip infusion time of SGN-35 is ≥30 min. Rapid intravenous injection or bolus administration should be avoided. SGN-35 should be administered using a special line for drip infusion. It must not be mixed with other drugs. When administering SGN-35, the line for drip infusion should be washed in physiological saline (Japan Pharmacopeia) before and after SGN-35 administration so that this drug may not be mixed with other drugs.

The dose of SGN-35 is calculated based on the body weight at the baseline, and expressed as an integral number by rounding the first decimal place. In subjects with a ≥ 10% change in the body weight during the study period, it should be regulated. In subjects weighing ≥100 kg, it should be calculated, regarding the body weight as 100 kg.

In this clinical study, 1.8 mg/kg of SGN-35 is administered to 6 children as Cohort 1. Based on the incidence of DLT during the DLT evaluation period, the tolerance of the dose/administration method is evaluated. If DLT is observed in ≤1 of the 6 children, 1.8 mg/kg of SGN-35 should be regarded as tolerable. On the other hand, if DLT is observed in 2 of the 6 children, a shift to Cohort 2 may be promoted. In Cohort 2, 3 subjects are added, and 1.8 mg/kg of SGN-35 is administered. If there is no DLT in the 3 subjects, 1.8 mg/kg of SGN-35 should be regarded as tolerable. However, if the third episode of DLT appears in Cohort 2, no patient will be newly registered.

### Dose-limiting toxicity (DLT)

DLT should be evaluated from the initial administration of this drug until administration on the first day of administration in Cycle 2. In subjects in whom the appearance of toxicity made this-drug administration in Cycle 2 impossible, and treatment was discontinued, DLT must be evaluated until safety follow-up.

Among adverse events of which the association with this drug cannot be ruled out, those corresponding to the following items are regarded as DLT. The grade is evaluated according to “Common Terminology Criteria for Adverse Events v4.03”:

- Grade 4 neutropenia persisting for ≥8 days
- Grade 3 febrile neutropenia requiring the administration of antibiotics
- Grade 4 febrile neutropenia
- Grade 4 thrombocytopenia

Grade 3 ≤ non-hematological toxicity. However, the followings are excluded:

- Grade 3 fatigue
- Grade 3 or 4 nausea (supportive therapy is permissible)
- Grade 3 or 4 vomiting (supportive therapy is permissible)
- Grade 3 abnormalities in non-hematological laboratory data showing recovery to grade 1 or a baseline condition (cases in which there were abnormalities at study enrollment) within 14 days
- Grade 3 or 4 allergic reactions

DLT will be finally evaluated by the trial-coordinating investigator.

### Follow-up

In subjects treated with this drug, the following assessments should be performed 28 days (7 days) after the final administration if it does not exceed the data cut-off day. These assessments should be conducted before the start of posttreatment. If examinations/observation/surveys are impossible for unavoidable reasons, such as complete withdrawal from this study, drop-out, death, and unfavorable conditions, the reasons must be recorded in original materials, and this study should be completed. In this case, deficits in examination/observation/survey items scheduled 28 days after the final administration are not regarded as deviations from the study protocol.

### Efficacy assessment method

The anti-tumor effects of SGN-35 are evaluated only in patients with measurable lesions according to the Revised Response Criteria for Malignant Lymphoma [13] based on the results of cervical, thoracic, abdominal, and pelvic computed tomography (CT) and positron emission tomography (PET), which were performed at the points established in the study protocol. At each point, the treatment response is assessed as CR, PR, SD, or PD. Lesions meeting the following criteria are regarded as measurable:

- Nodular masses of lymph nodes or extranodular organs evaluated as lymphoma on CT
- The lesion can be clearly measured in two intersecting directions on cross sections of CT.
- The maximum diameter exceeds 1.5 cm on cross sections of CT.
- Positive findings on FDG-PET.

Among measurable lesions, 6 lesions at maximum in the order of maximum diameter on cross sections of CT should be selected as target lesions regardless of nodular or extranodular lesions.

If subjects are positive for bone marrow infiltration on baseline assessment, follow-up by bone marrow aspiration or biopsy may be necessary. To evaluate the treatment response as CR, subjects must be negative for bone marrow infiltration. If morphological examination-based evaluation is impossible, the results of examination using immunostaining should also be considered.

### Statistical analysis

#### Analysis set

Patients enrolled in this study and treated with the investigational drug at least one session are regarded as a full analysis set (FAS). However, patients who were shown to violate the study protocol or GCP after enrollment and those who were considered ineligible after enrollment should be excluded from FAS. Safety analysis set is defined as patients enrolled in this study and treated with the investigational drug at least one session. DLT analysis set is defined as the following subjects:

- Subjects with ≥1 DLT during the DLT assessment period (from initial administration until administration in Cycle 2)

or

- Those to whom an established dose of SGN-35 was administered in Cycle 1, and in whom observation for DLT assessment was completed.

Pharmacokinetics (PK) analysis set is defined as patients enrolled in this study and observed at least one PK data.

### Safety endpoints

The incidence of DLT will be calculated in DLT analysis set. The incidence of adverse events/reactions to the investigational drug will be calculated with respect to events, severity, and grade with safety analysis set.

### Efficacy endpoints

The ORR, CR rate, and their 95% confidence interval will be calculated in the FAS. To estimate the response duration, PFS, and EFS, the Kaplan-Meier method will be used. Their 95% confidence interval will be calculated with Greenwood’s formula.

### Interim analysis and monitoring

As it may be difficult to obtain sufficient information useful for efficacy assessment during this clinical study, interim analysis for efficacy assessment will not be conducted.

To confirm that this clinical study is being safely and adequately conducted according to the study protocol and relevant regulations, and that data reliability is sufficiently secured, hospital visit monitoring with direct reading, including the comparison of case reports with original materials by the monitoring director or persons in charge of monitoring, will be performed. The principle investigator/investigators must accept hospital visit monitoring by the monitoring director and persons in charge of monitoring designated before the start of this study, and provide all study-associated records, such as original materials, for direct reading.

When there is an inconsistency between original materials and case reports, the monitoring director or persons in charge of monitoring must obtain records explaining its reasons from the chief investigator.

### Pharmacokinetics

In a PK analysis set, pharmacokinetic parameters (Cmax, AUC, etc) for serum concentration of the drug will be estimated using non-compartmental analysis. Similarly, the serum concentration of all antibodies and plasma concentration of MMAE will be analyzed. Blood specimens used for evaluation of pharmacokinetics are collected at the time shown in Table 1.

**Table 1:** Timing of sample collection for pharmacokinetics assessment

Cycle	Date	Time	Permissible range
Cycle 1	Day 1	Before administration	≤ 2 h.
		10 min after the completion of administration	± 5 min.
	Day 2	24 h after the completion of administration	± 1 h.
	Day 4	72 h after the start of administration	± 2 h.
	Day 8	168 h after the start of administration	± 3 h.
	Day 15	336 h after the start of administration	± 3 h.
Cycle 2	Day 1	Before administration	≤ 2 h.
		10 min after the completion of administration	± 5 min.
	Day 2	24 h after the completion of administration	± 2 h.
	Day 4	72 h after the start of administration	± 2 h.
	Day 8	168 h after the start of administration	± 3 h.
	Day 15	336 h after the start of administration	± 3 h.
Cycle 3	Day 1		≤ 2 h.
–	Safety follow-up		

Blood samples for pharmacokinetics assessment should be collected at the following points. In subjects in whom blood collection is considered difficult, blood samples may be collected before administration in each cycle

## Discussion

Pediatric patients should be administered medicines that have been appropriately evaluated for their use. In the past, however, new approved drugs have been used for pediatric patients without assessment of the efficacy and safety in those patients. Clinical studies involving adults with recurrent or refractory HL or ALCL in Japan and other countries demonstrated the efficacy and safety of SGN-35 [10, 11]. The present study will prove the efficacy and safety of SGN-35 in Japanese children.

## Abbreviations

ADC: Antibody-drug conjugate
ALCL: Anaplastic large-cell lymphoma
ALT: Alanine aminotransferase
AST: Aspartate aminotransferase
CR: Complete remission
CT: Computed tomography
DLT: Dose-limiting toxicity
EFS: Event-free survival
FAS: Full analysis set
G-CSF: Granulocyte-colony stimulating factor
HBs: Hepatitis B virus surface
HL: Hodgkin’s lymphoma
LD-IFRT: Low-dose involved field radiotherapy
MMAE: Monomethylauristatin E
MTD: Maximum tolerance dose
NYHA: New York Heart Association
ORR: Overall response rate
PD: Progressive disease
PET: Positron emission tomography
PFS: Progression-free survival
PK: Pharmacokinetics
PR: Partial remission
SALCL: Systemic ALCL
SG: Inc., Seattle Genetics, Inc.
ULN: Upper limit of normal
